# Is the bioactivity of induced membranes time dependent?

**DOI:** 10.1007/s00068-021-01844-4

**Published:** 2021-12-06

**Authors:** Jan Gessmann, Thomas Rosteius, Hinnerk Baecker, Kavitha Sivalingam, Elvira Peter, Thomas Armin Schildhauer, Manfred Köller

**Affiliations:** 1 Department of General and Trauma Surgery, BG University Hospital Bergmannsheil, Ruhr-University Bochum, Bürkle-de-la-Camp-Platz 1, 44789 Bochum, Germany; 2 Department of Surgical Research, BG University Hospital Bergmannsheil, Bürkle-de-la-Camp-Platz 1, 44789 Bochum, Germany

**Keywords:** Masquelet, Induced membrane, Bioactivity, Bone defect, Mesenchymal stem cells

## Abstract

**Purpose:**

The induced membrane technique (IMT) is a two-stage surgical procedure for reconstruction of bone defects. Bone grafting (second stage of IMT) is recommend after 4–8 weeks assuming the highest bioactivity of IMs. However, larger studies concerning the biology and maturation of IMs and a potential time dependency of the bioactivity are missing. Therefore, aim of this study was the time-dependent structural and cellular characterization of cement spacer IMs concomitantly to an analysis of membrane bioactivity.

**Methods:**

IMs from 60 patients (35–82 years) were obtained at different maturation stages (1–16 weeks). IMs were studied by histology and co-culture with mesenchymal stem cells (MSC). IM lysates were analyzed by ELISA and protein microarray.

**Results:**

Increasing vascularization and fibrosis were found in membranes older than 4 and 7 weeks, respectively. MSC grew out from all membranes and all membranes enhanced proliferation of cultured MSC. Osteocalcin and osteopontin (in membrane lysates or induced in MSC by membrane tissue) were found over all time points without significant differences. In contrast to alkaline phosphatase activity, increasing levels of osteoprotegerin were found in membranes.

**Conclusion:**

The histological structure of IMs changes during growth and maturation, however, biologically active MSC and factors related to osteogenesis are found over all time points with minor changes. Thus, membranes older than 8 weeks exert regenerative capacities comparable to the younger ones. The postulated narrow time frame of 4–8 weeks until bone grafting can be questioned and surgeons may choose timing for the second operation more independently and based on other clinical factors.

## Introduction

The induced membrane technique (IMT) is a two-stage surgical reconstructive procedure for segmental bone defects. Since the first description by Masquelet et al. [1], the technique has gained increasing popularity as a simple and less complex alternative to distraction osteogenesis. Despite many reported studies concerning the biological role of the induced membrane, many questions still remain on cornerstones of the procedure such as ideal fixation, cement spacer composition and timing of bone grafting [2–5].

The general concept of the technique is based on the generation of membrane formed around a cement spacer that is implanted during the first surgical step. The spacer is removed during the second surgical step and the cavity is filled with cancellous bone graft. Thereby, the membrane is thought to serve not only as a mechanical and shaping protective covering for the cancellous bone graft but to actively influence bone healing with the secretion of osteoinductive and angiogenetic growth factors and in containing mesenchymal stem cells [2, 6]. Basic research of animal and very few human membranes found the highest osteoinductive bioactivity after 2–4 weeks suggesting bone grafting after this short time interval. However, clinical, not evidence-based recommendations range from 4 to 8 weeks but there are successful treatment reports when bone grafting was performed several months or even years after cement spacer implantation [7–11]. This large variation in timing and the apparent contradiction between basic research results and the actual clinical procedure underline that the biological mechanisms of the technique still remain relatively unclear [4].

Therefore, the aim of this study was the structural and cellular characterization of cement-spacer-induced membranes and to analyze a possible time dependence of the bioactivity. The results should support surgeons in the decision making when to perform the bone-grafting step of the IM procedure.

## Methods

### Patients and membrane specimen

The study was reviewed and approved by the local ethical committee (registration number (16-5673). All procedures were performed in accordance with the ethical standards of the institutional research committee and with the 1964 Declaration of Helsinki and its later amendments.

**Fig. 1:**
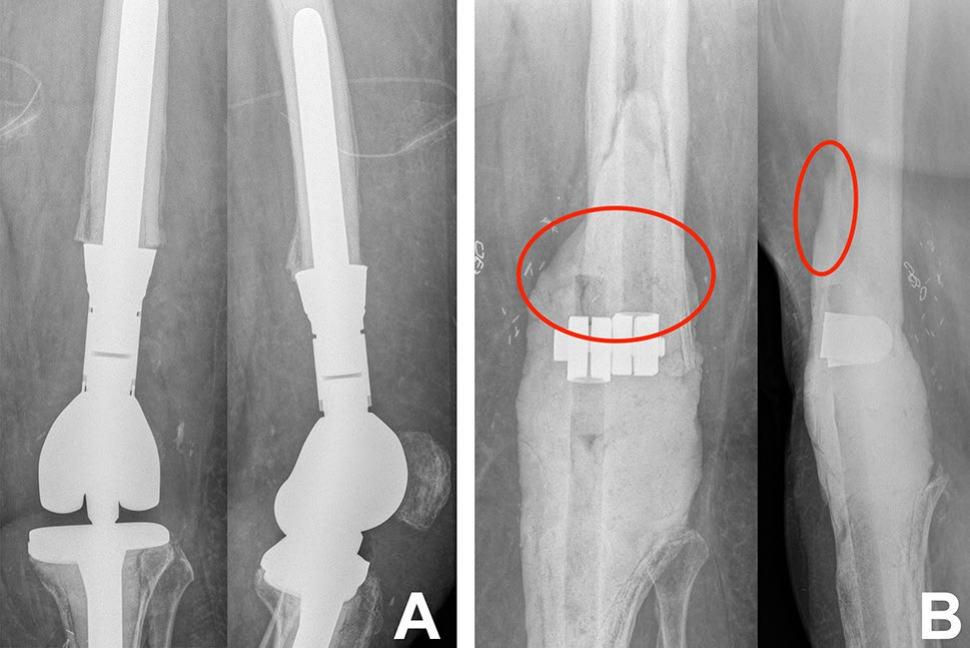
Female patient with periprosthetic joint infection (PJI) of a revision TKA and severe bone loss of the distal femur. Before (**A**) and after removal of the infected TKA, radical debridement and implantation of the PMMA-spacer (**B**). The red circle demonstrates the site at which the membrane samples were collected

**Fig. 2:**
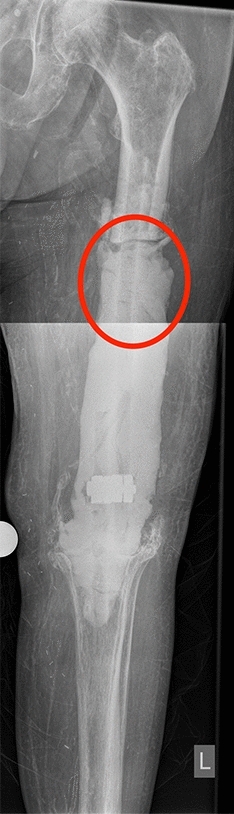
Male patient undergoing two-stage exchange surgery due to PJI of a revision TKA and severe bone loss of 2/3 of the femur diaphysis. The red circle demonstrates the site at which the membrane samples were collected

Membranes of 60 patients (Table 1) were harvested during the removal of polymethyl methacrylate (PMMA) cement spacers (fixed between distal femur and proximal tibia) at the second stage of revision total knee arthroplasty (TKA) or joint arthrodesis due to large femoral bone defects. This procedure is equivalent to the two-stage Masquelet technique for segmental bone defect repair. All membrane samples were taken from patients with large bone defects and with a safe distance to any joint structures (Figs. 1, 2). While, during the Masquelet technique, the induced membrane has to be preserved, here, membranes are completely removed prior to the implantation of the revision TKA or arthrodesis. Immediately after removal, membrane samples were prepared for the different analyses.

**Table 1:** Patient characteristics

	Age (years) Mean ± SD	Gender (*n*)	Spacer-retention period (days) Mean ± SD
Total *n* = 60	63 ± 14	Female: 28 Male: 32	47 ± 21
Group 1 *n* = 10	54 ± 14	Female: 5 Male: 5	19 ± 5
Group 2 *n* = 26	63 ± 13	Female: 15 Male: 11	41 ± 5
Group 3 *n* = 17	69 ± 12	Female: 5 Male: 12	55 ± 4
Group 4 *n* = 7	65 ± 18	Female: 3 Male: 4	91 ± 16

The obtained data were grouped into four time interval groups, according to the duration of PMMA spacers at the surgical site: group 1 = 8–28 days, group 2 = 29–49 days, group 3 = 50–63 days, and group 4 = 78–113 days. Group-related patient characteristics are summarized in Table 1.

The spacers were custom-made for each patient using PMMA loaded with antibiotics according to the specific antibiogram. Vancomycin was added in 55 of all the cases, vancomycin/amphotericin was used in 2 cases, gentamicin in 2 cases, and teicoplanin/anidulafungin in 1 case.

### Outgrowth of cells from membranes

Membrane pieces (size 4–5 mm) were transferred into 75 cm^2^ cell culture flasks (BD Falcon) containing RPMI/FCS (RPMI1640 from ThermoScientific with 10% fetal calf serum, FCS, from Sigma Aldrich) and cultured for 14 days with medium exchange every 3–4 days. Outgrown cells were harvested (0.05% Trypsin/EDTA) and suspended in cell culture medium (RPMI/FCS). After 1–2 cell passages, the expression of CD90 and CD105 was analyzed by immunohistochemistry (see below). In addition, cells were analyzed for their potential to differentiate towards the osteogenic, adipogenic and chondrogenic lineages after culture in respective differentiation media (ThermoScientific) for 3 weeks.

### Histological analysis

Membrane pieces were processed for histological and immunohistological analyses (Institute of Pathology) as 5 µm thick slices. Histological stainings were performed by hematoxylin/eosin (H&E staining) and Elastica van Gieson stainings (EvG).

For immunohistochemistry, heat-based antigen recovery was performed using a recovery solution (Vector Lab, Burlingame, CA, USA) at 121 °C for 10 min (Systec CDX-23 autoclave, Systec GmbH, Linden, Germany). Endogenous enzyme activity was blocked with Vector BLOXALL blocking solution (BIOZOL, Eching, Germany) for 10 min. Subsequently sections were washed in PBS/1% BSA for 5 min. Antibodies (CD, 34, CD90, CD105) were from Bio-Techne GmbH (Wiesbaden, Germany).

Antibody binding was visualized using the Vectastain ABC-AP kit (Vector) according to the manufacturer’s protocol. Stained membrane sections or cell cultures were analyzed by microscopy (BX61, Olympus, Hamburg, Germany) and images were taken using a digital camera (DP80, Olympus).

### Analysis of membrane bioactivity using direct or indirect co-culture with mesenchymal stem cells (MSC)

Membranes were co-cultured in direct or indirect contact with growing MSC (Lonza, Walkersville, MD, USA). MSC were plated at a density of 1 × 10^4^ cells/ml RPMI/FCS in 24-well cell culture plates and cultured overnight. Subsequently, medium was renewed and punched-out pieces (ø 5 mm) were added either directly onto the growing MSC layer or were placed in transwell inserts (0.2 µm pore size) as indirect co-culture. To determine osteopromotive effects of membrane pieces, the supernatants were analyzed after 2 weeks of co-culture for C-terminal propeptide of procollagen I (CICP), osteoprotegerin (OPG), osteocalcin (OC) and bone-specific alkaline phosphatase (BAP) by ELISA (TECOmedical GmbH, Bünde, Germany). The proliferation of MSC was analyzed using BrdU ELISA (Roche Diagnostic Mannheim, Germany) according the manufacturer’s protocol.

### Protein-microarray

Membrane biopsies were punched out using an 8 mm biopsy punch (kai Europe GmbH, Solingen, Germany) and were homogenized in 1 ml lysis solution (Sigma, Taufkirchen, Germany). Lysates were centrifuged at 10000×*g* at 4 °C for 10 min and stored at − 70 °C. Quantification protein content was performed using Pierce BCA protein assay kit (Thermo Fisher Scientific) according to the manufacturer’s instruction and by a plate-reader (MRX Revelation, Dynex Technologies, Denkendorf, Germany).

Lysates were analyzed using the chemiluminescence-based Proteome Profiler Array Human XL Cytokine Array Kit (Bio-Techne GmbH) according to manufacturer’s instruction. Chemiluminescence images (Fig. 1) were taken by a CCD-Imager (Amersham Imager 600 RGB, GEHealthcare Life Sciences). The obtained dot signals (gray values) were quantified using ImageQuant TL 8.1 software (GEHealthcare Life Sciences). The calculated values obtained from individual lysates were adjusted according to protein content. In addition, single factors within membrane lysates were analyzed by ELISA (TECOmedical, see above).

### Statistical analysis

Statistica 13.0 software was used for statistical analysis of data. Membrane group differences were calculated using one-way analysis of variance (ANOVA) with correction for multiple comparisons (Bonferroni). To analyze a correlation between bioactive factors and the age of the membranes, Spearman rank correlations were calculated. A *p* value ≤ 0.05 was considered statistically significant.

## Results

### Histological analysis of induced membranes at different time points

**Fig. 3:**
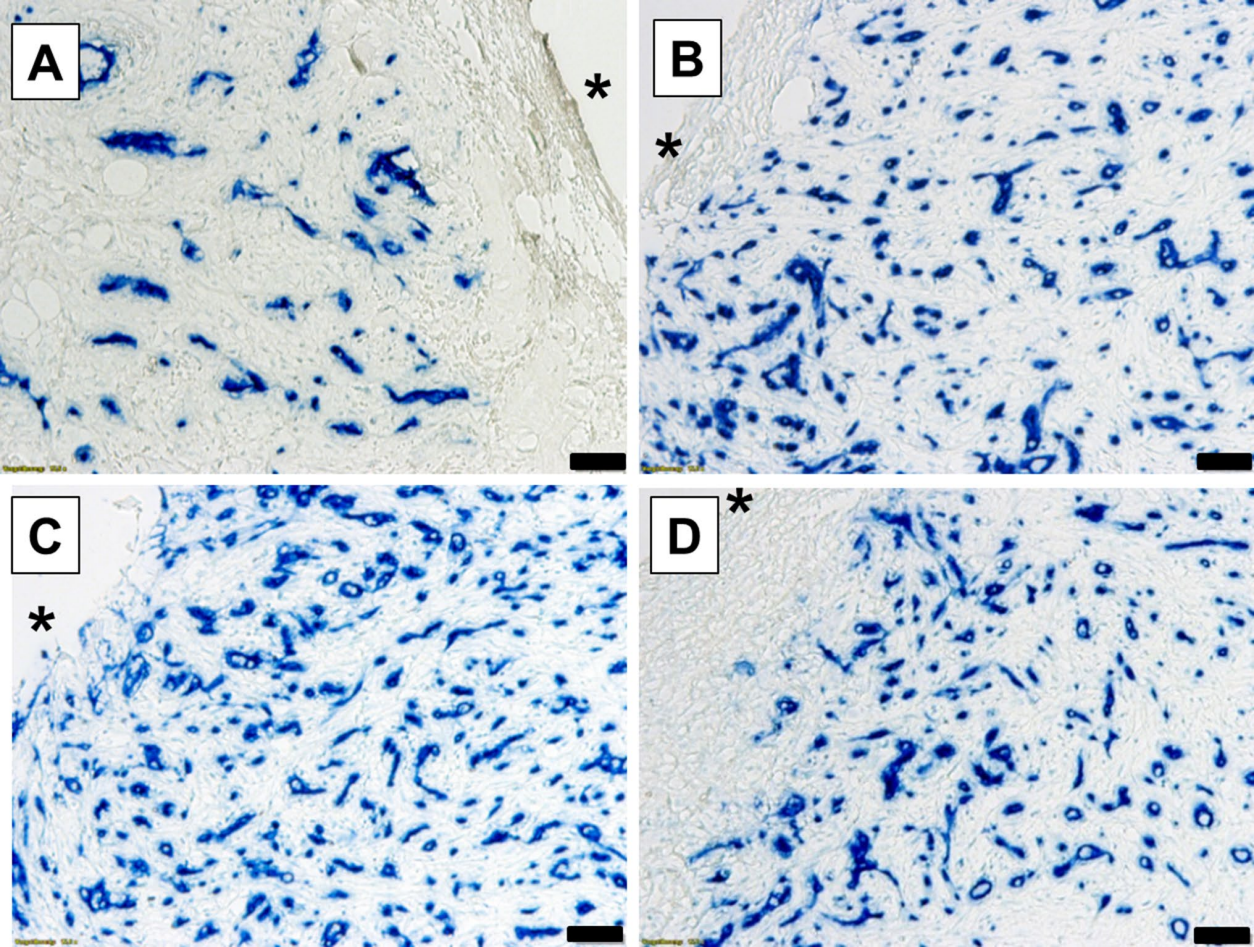
Identification of vascular tissue of induced membranes by CD34 expression (immunohistochemistry) over time (**A** group 1: 21 days; **B** group 2: 46 days; **C** group 3: 56 days; and **D** group 4: 83 days. Black stars (*) indicate the position of the PMMA-spacer before removal. Blue structures represent CD34-positive cells. Scale bars, 50 µm

**Fig. 4:**
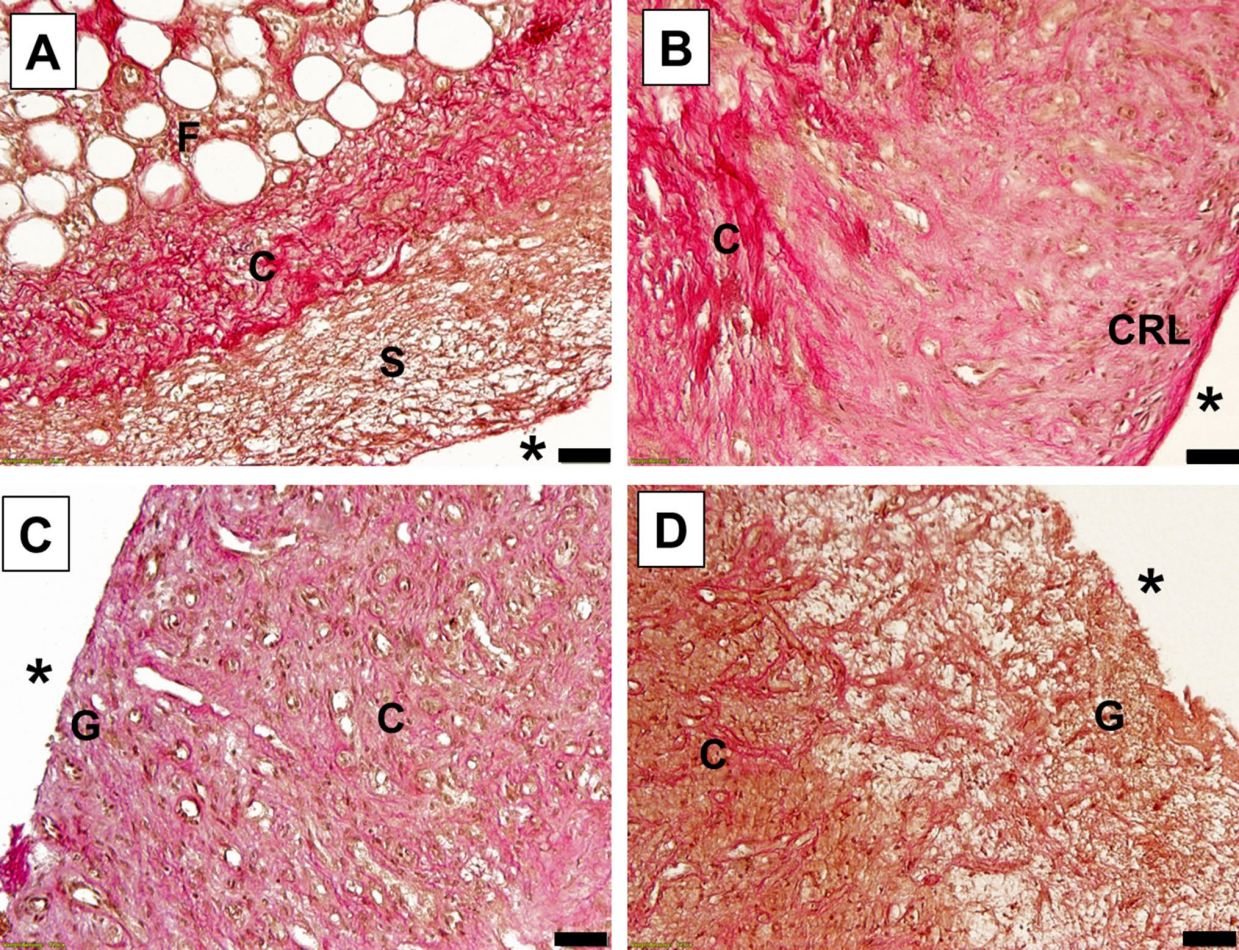
Representative paraffin sections of induced membranes (Elastica van Gieson staining, EvG) demonstrating their architectural organization and their histological changes over time (**A** group 1: 9 days; **B** group 2: 24 days; **C** group 3: 42 days; and **D** group 4: 78 days. Black stars (*) indicate the position of the PMMA-spacer before removal. Black letters indicate tissue types: C, collagen fibers; CRL, cell-rich layer; F, fatty tissue; G, granulation tissue, S, stromal components. Scale bars, 50 µm

In general, the induced membranes showed characteristics of a low-grade infection according to a Krenn and Morawietz grade 2 [12] with individually variable signs of inflammation such as leukocyte infiltration. The membranes appeared manifold vascularized which was increased in the later time groups (Fig. 3). The histological structure of the membrane was two- or three-layered and changed over time. Collagenous structures increased over time as is exemplarily shown in Fig. 4. Highly fibrosed connective tissue (Fig. 4C) or fiber-rich granulation tissue (Fig. 4D) is found at later time points.

### Analysis of biological activity of induced membranes

Outgrow of spindle-like cells occurred from all membranes after culture of membrane pieces. Expanded outgrown cells were positive for CD90 and CD105 and were able to differentiate into adipogenic, osteogenic and chondrogenic lineages (data not shown) which indicate that all induced membranes contained mesenchymal stem cells (MSC). This outgrow assay did not allow to quantify the primary MSC content of the membranes. Nevertheless, this differentiation capacity of outgrown cells corresponds to a general capacity of membranes to promote tissue regeneration including osteogenesis even at later time points.

The biological activity of the membranes was analyzed using co-culture with mesenchymal stem cells and by factor analysis of membrane lysates (protein microarray and ELISA).

### Analysis of factors related to osteogenesis within membrane lysates

**Fig. 5:**
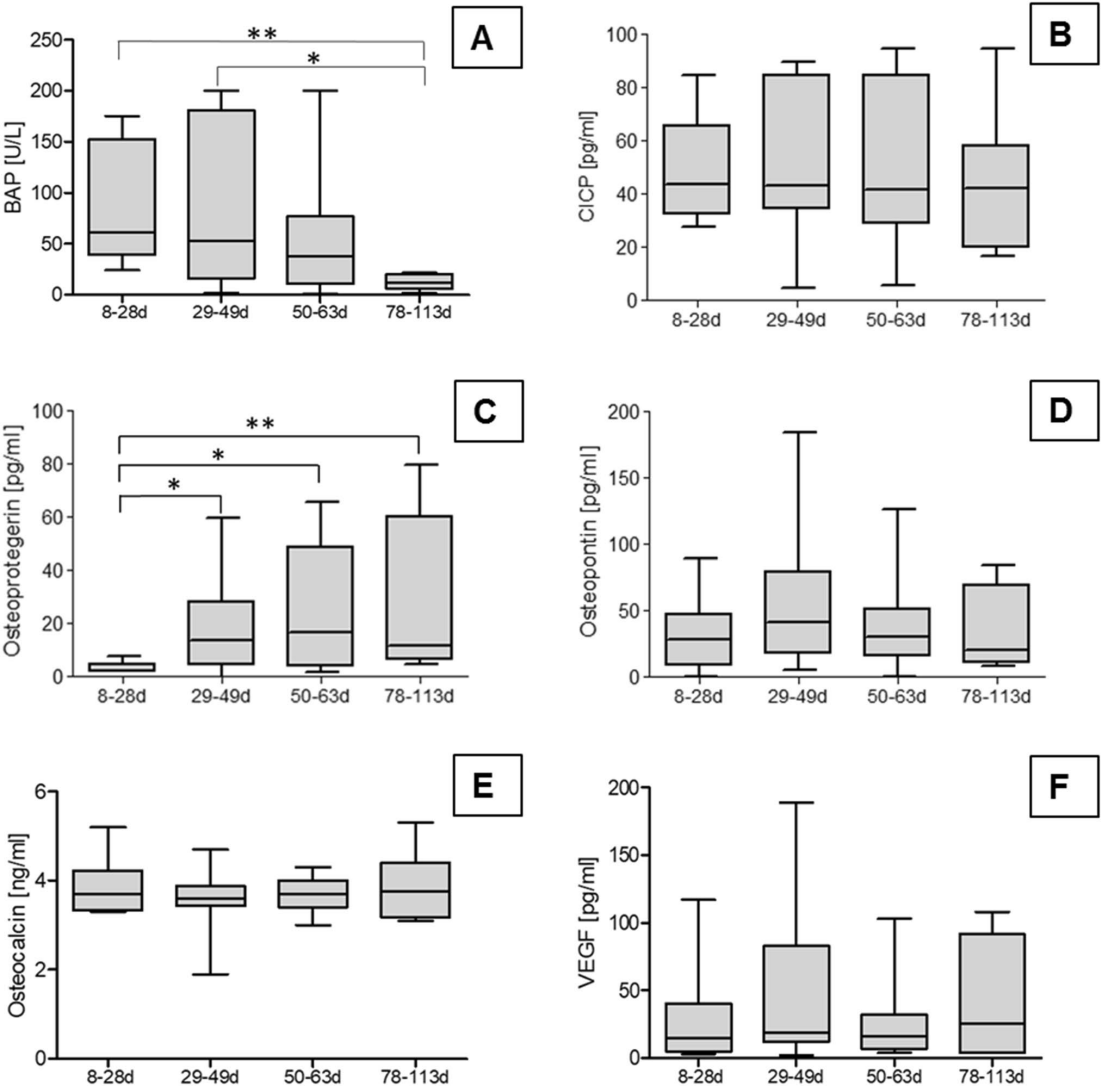
Quantification of bioactive factors in lysates of membranes removed at different times by ELISA. **A** Bone-specific alkaline phosphatase activity (BAP). **B** Carboxy-terminal propeptide of type I collagen (CICP). **C**, Osteoprotegerin (OPG). **D** Osteopontin. **E** Osteocalcin. **F** Vascular endothelial growth factor (VEGF). Data represent values given as box whiskers plots. The box represents 25th to 75th percentile of data distribution, the median is given by the horizontal line. Whiskers represent minimal and maximal values. Membrane removal time is given as days (d)

To determine osteogenesis-related factors within the membrane tissues respective lysates were analyzed. The concentrations of CICP, osteocalcin and osteopontin did not differ significantly among membrane time groups (Fig. 5B, D, E). Comparable results were obtained for the concentrations of VEGF (Fig. 5F). In contrast, the activity of BAP decreased and concentrations of osteoprotegerin increased over time (Fig. 5A, C). These differences may be related to changes in membrane histology during maturation.

The use of the protein microarray allowed the detection of multiple factors such as cytokines in a single-membrane lysate. Analysis of the array readouts revealed that many factors of the used array gave no or only faint signals. Thus, only factors with sufficient expression and relevance to angiogenesis, inflammation, or tissue regeneration are presented in Table 2. No significant differences in expression of the enlisted bioactive factors were detected with exception of IL-8 which was found increased in the later time group 3. In addition, there were no statistically significant correlations between the expression of the different bioactive factors in Table 2 and the age of the membranes.

**Table 2:** Expression of bioactive factors in membrane lysates analyzed by protein microarray

Factor	Biological activity	Group 1 (median)	Group 2 (median)	Group 3 (median)	Group 4 (median)
Angiogenin	A	106	102	132	97
Angiopoietin-2	A	9	7	8	6
Complement component C5/C5a	I	9	7	9	10
IFN-γ	I	4	3	3	3
C-reactive protein	I	82	93	74	79
ENA-78 (CXCL5)	I	10	11	15	7
IL-1ra	I	10	10	7	8
IL-8 (CXCL8)	I	24	21	33^a^	20
IL-17A	I	7	6	7	7
MCP-1 (CCL2, MCAF)	I	9	9	9	11
MIF	I	40	38	42	27
RANTES (CCL5)	I	14	13	18	14
Chitinase 3-like 1	R	95	87	90	83
Endoglin (CD105, ENG)	R	77	71	75	67
FGF basic	R	3	3	3	5
IGFBP-2	R	6	8	11	7
Osteopontin (OPN)	R	77	73	100	69

Factors were roughly allocated to their main biological activity: angiogenic (A), inflammatory (I), or involved in tissue regeneration (R). Data represent chemiluminescence signal intensities given as median values of respective time groups. Differences of factors in the time groups 1–4 were calculated using one-way analysis of variance (ANOVA) with correction for multiple comparisons (Bonferroni) (*p* ≤ 0.05)

^a^Statistical significance between group 3 and group 1, 2 and 4

### Influence of induced membrane tissue with different retention times on the proliferation of MSC

**Fig. 6:**
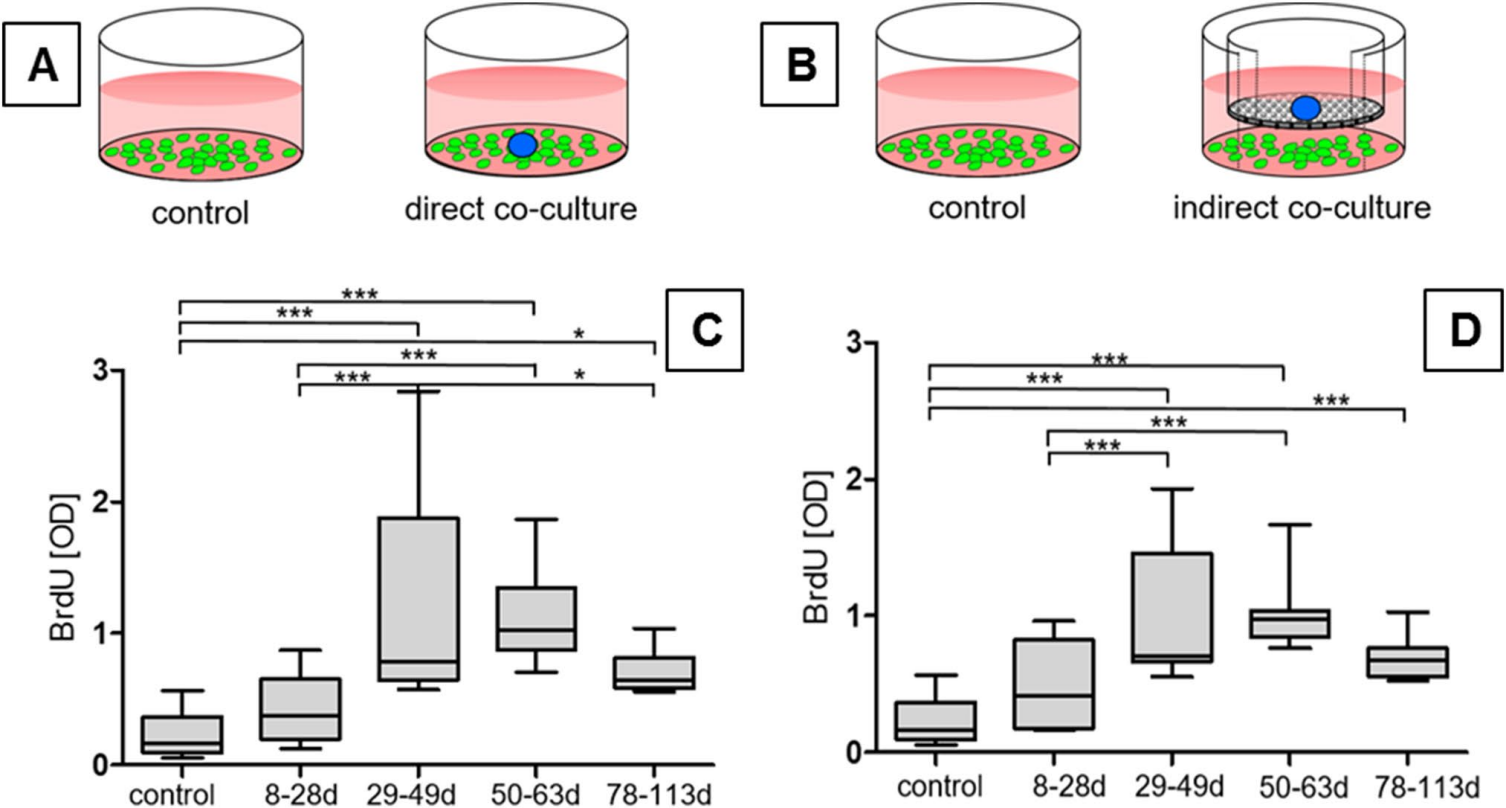
Effects of direct and indirect co-cultures of membranes removed at different times on MSC proliferation after incubation for 7 days. **A** Experimental direct co-culture setup. **B** Experimental indirect co-culture setup. Green, subconfluently growing MSC; blue, added membrane piece. Proliferation of MSC measured by BrdU assay (**C** direct co-culture; **D** indirect co-culture). Data represent the proliferation (OD values) given as box whiskers plots. The box represents 25th to 75th percentile of data distribution, the median is given by the horizontal line. Whiskers represent minimal and maximal values. Membrane removal time is given as days (d), controls represent MSC without added membranes

The proliferation-inducing capacity of membranes was analyzed by addition of membranes to subconfluently growing MSC using co-culture models (Fig. 6A, B). As is shown in Fig. 6C, D, the membranes from all time groups enhanced the proliferation of MSC. However, especially membranes of the later time groups (2–4, > 28 d) enhanced proliferation of MSC groups compared to membranes belonging to group 1 (− 12 d). Among direct and indirect co-culture, no differences in the promotion of MSC proliferation were observed.

### Analysis of factors related to osteogenesis using co-culture models

**Fig. 7:**
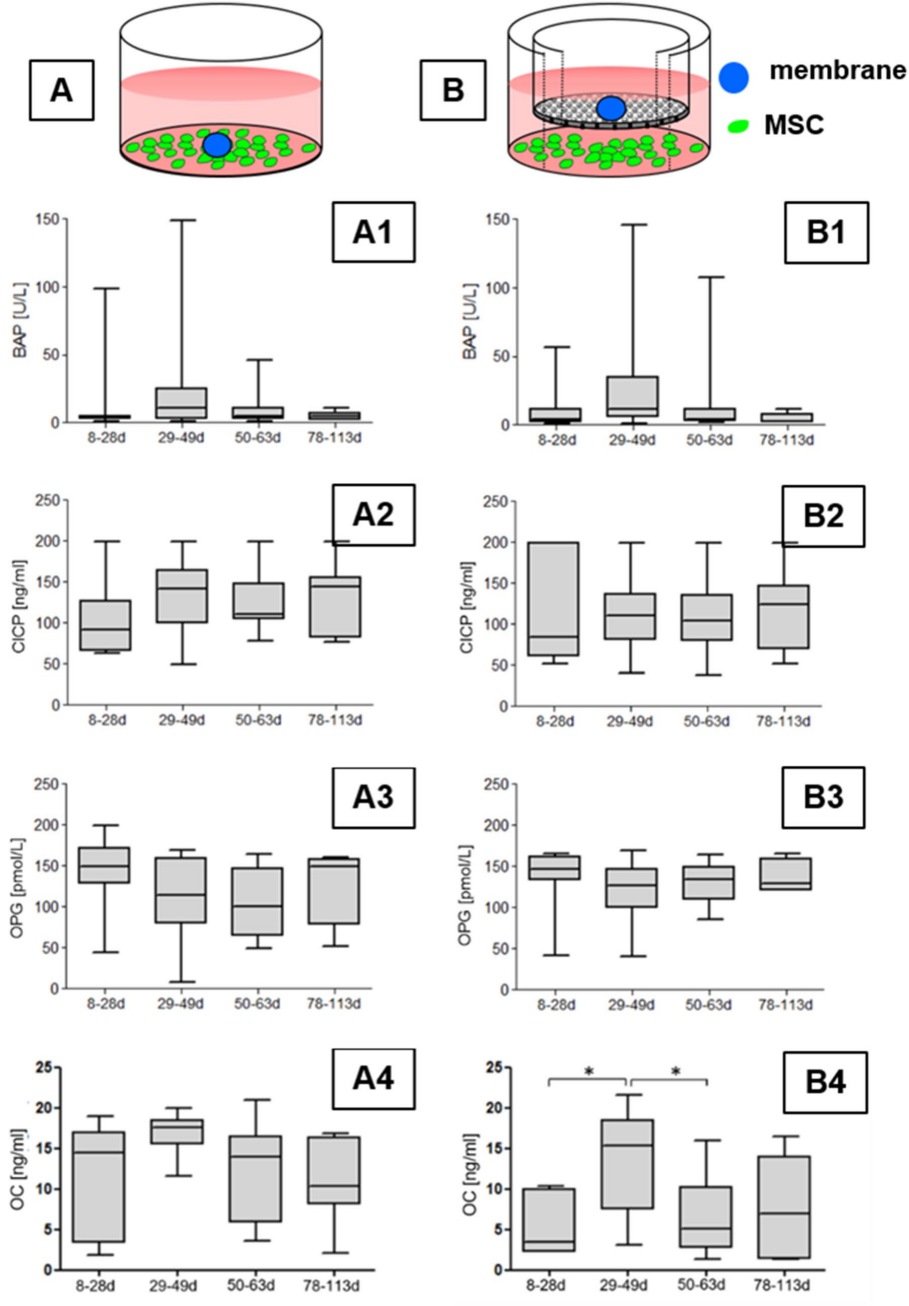
Effects of direct and indirect co-cultures of membranes removed at different times with MSC on the expression of osteogenic factors analyzed by ELISA. Diagrams of experimental setups (**A** direct co-culture; **B** indirect co-culture). Green, subconfluently growing MSC; blue, added piece of membrane. **A1** and **B1** Bone-specific alkaline phosphatase activity (BAP). **A2** and **B2** Concentration of carboxy-terminal propeptide of type I collagen (CICP). **A3** and **B3** Concentration of osteoprotegerin (OPG). **A4** and **B4** Concentration of osteocalcin (OC). Data represent values given as box whiskers plots. The box represents 25th to 75th percentile of data distribution, The median is given by the horizontal line. Whiskers represent minimal and maximal values. Membrane removal time is given as days (d)

Osteogenesis-related factors were analyzed in supernatants of the co-cultures (Fig. 7A, B). Significant differences in the activities (BAP) or concentration (CICP, OPG) of measured factors were not observed with exception of osteocalcin using the indirect co-culture model (Fig. 7B4) but values obtained from the corresponding direct co-culture model (Fig. 7A4) did not show comparable differences. However, the median value of osteocalcin concentrations was the highest in time group 2.

## Discussion

Since the introduction of the induced membrane technique (IMT) by Masquelet et al. [1] for the treatment of large bone defects, IMT has been proved its clinical success and is now well accepted and widely used [4, 13]. Experimental studies and ongoing clinical practice are directed to further improvements of IMT [5, 14, 15]. At present, the optimal time frame for the second-stage operation is a controversial issue. The commonly recommended 4–8 weeks are based mainly on clinical experiences and the level of evidence according to Wright [16] has been graded level B recently [13]. The recommendation is supported by animal models and induced membrane parameters such as vitality, thickness, and expression of growth factors [13, 17–19]. In clinical practice, this time window is not always possible to meet especially in cases of prolonged soft tissue healing or free flap surgery confronting surgeons with the problem whether to perform bone grafting at later time points or to exchange the spacer to induce new membrane [11]. However, in the literature, there are successful bone reconstructions reported with a time interval of several month and even years between cement spacer implantation and bone grafting [7–11, 20, 21].

Obviously, an optimal timing of the second stage is also dependent on the maturation of the induced membrane. Our study includes also membranes which developed longer than 8 weeks prior to removal (about 40% of all samples) and we included membranes from 60 patients which is the largest number to our knowledge. Although a two-staged revision TKA after PJI is not a bone reconstruction procedure, large bone defects at proximal tibia and distal femur sites, which extend beyond the anatomical region of the knee (Figs. 1, 2), resemble large segmental bone defects that are filled with a cement spacer. Furthermore, the Masquelet technique has been described not only for diaphyseal bone defects, but also for arthrodesis cases in which the previous joint space is filled with a cement spacer after radical debridement [22–24]. In contrast to a Masquelet procedure in which the preservation of the membrane is mandatory, the induced membranes in our study were completely available for all analyses eliminating the problem of too small sample sizes like in other studies [6].

The tissue structure of the membranes and cell-compositional changes during membrane maturation are well described and correspond to the histologic analyses of our study. Generally, induced membranes consist of a fibroblast/collagen-based matrix with an inner synovial-like epithelium and an outer well-vascularized layer [7, 18, 19, 25, 26]. Induced membranes also contain invaded leukocytes, osteoclasts, and are rich in mesenchymal stem cells [26, 27]. The biological properties of the induced membrane to favor bone formation are most likely related to different paracrine factors and the timing of their release. Angiogenic and osteogenic factors such as VEGF, TGF-ß1, BMP-2, or RUNX2 have been found in induced membranes [28–31]. Typically, angiogenic and endothelial-related factors (VEGF) can be detected at early phases of membrane development and decreased subsequently [6, 18, 19, 32]. Our study revealed no significant differences in VEGF concentrations, however, membranes showed histologically an increase in vascularization over time.

Besides the analysis of factors within membranes, paracrine effects of membrane pieces on MSC were also measured by co-culture models. Such co-cultures have also been used by others; however, mainly protein extracts from membranes were used there instead of membrane pieces [6, 18, 29, 32, 33]. Aho et al. described enhanced calcium deposition and PINP (N-terminal propeptide of type I collagen) levels in co-cultures of MSC with induced membranes and found that 1-month-old membranes induced higher calcium deposition and PINP production compared to 2-month-old membranes [6]. In our study, we analyzed CICP (C-terminal propeptide of type I collagen) whose concentration is also indicative for collagen production and detected elevated CICP levels in co-cultures of MSC with added membranes in later time groups 2, 3 and 4. We found a trend for enhanced alkaline phosphatase activity in MSC due to the presence of membranes which correlate to the study of Aho et al. and other reports on co-cultures using membrane lysates [6, 18, 29, 32, 33].

An enhancement of MSC proliferation in the presence of induced membranes was also observed by others using induced membrane protein extracts [18, 32]. However, our experimental model (co-culture of induced membrane piece MSC without osteogenic medium) did not reveal detectable synthesis of calcium rich extracellular matrix which was described due to the use of an osteogenic medium [29]. Gindraux et al. [7] also observed that MSC cultured with induced membranes needed an osteogenic medium for enhanced calcium deposition.

Our study revealed that the kind of method used for the analysis of bioactive factors partly influenced the results with respect to single factors. We detected decreasing BAP expressing in membrane lysates over time but not in the co-culture models where the BAP concentrations in supernatants were not different among the time groups. OPG expression was increased over time in membrane lysates but not in supernatant of co-culture models. Such differences are clearly related to the different experimental setups. Observed differences of the kinetics between osteoprotegerin and alkaline phosphatase activity in membrane lysates may be related to changes in membrane histology during maturation.

In general, the histological and biochemical characteristics of the induced membranes found by our study resemble various comparable reports using animal models. However, our in vitro results did not support a postulated narrow time frame (4–8 weeks) for the second-stage procedure but let us hypothesize that the time gap between first- and second-stage procedures might be extended. Recently, it was shown by clinical studies that waiting longer between the two surgeries did not delay bone union [7, 11].

Beside the optimal time frame for staging other factors will clearly influence the bioactivity of induced membranes such as anatomical site, soft tissue environment, or the nature of the bone defect or patient variables [13]. Nevertheless, more clinical data are necessary to confirm the presented in vitro results.
